# The Impact of MTHFR 1298 A > C and 677 C > T Gene Polymorphisms as Susceptibility Risk Factors in Cervical Intraepithelial Neoplasia Related to HPV and Sexually Transmitted Infections

**DOI:** 10.1007/s13224-020-01363-z

**Published:** 2020-08-24

**Authors:** Amir Sohrabi, Fatemeh Bassam-Tolami, Mohsen Imani

**Affiliations:** 1 Department of Medical Epidemiology and Biostatistics, Karolinska Institutet, Nobels väg 12A, Solna Campus, PO Box 171 77, Stockholm, Sweden; 2 Department of Biology, Tehran Shargh Branch, Payame Noor University, Tehran, Iran; 3 Department of Molecular Biology, Research Center of Health Reference Laboratory, Ministry of Health and Medical Education, Tehran, Iran

**Keywords:** HPV, STI, Genital infection, Cervical cancer, MTHFR, Polymorphism

## Abstract

**Background:**

HPV genotypes are the most common etiological factor for genital neoplasia. It would appear that sexually transmitted infections accompanied with HPV genotypes might have synergistic interactions in cancer progression. The genetic polymorphisms are involved in metabolizing carcinogens which may contribute to the susceptibility of developing genital cancers by less efficient or overly down metabolic pathways and cell signaling. MTHFR polymorphisms are related to several metabolic disorders and human cancers. We investigated the contribution of MTHFR 1298 and MTHFR 677 polymorphisms as potential risk factors for outcomes with HPV genotypes and STIs in Iranian population.

**Materials and Methods:**

As a case–control study, MTHFR A1298C and C677T were assessed for SNPs analysis using a PCR–RFLP assay in 50 cervical intraepithelial neoplasia (CIN) cases, 98 HPV-positive subjects and 47 non-cancerous/non-HPV patients as healthy controls.

**Results:**

Finding suggested a significant association between the MTHFR 1298 CC polymorphisms (OR = 3.5, 95% CI = 1.13–10.82, *P* ≤ 0.05) in women with CIN as compared to non-cancerous/non-HPV subjects. There was not a significant difference of MTHFR 677 between outcomes.

**Discussion:**

It would seem MTHFR 1298 CC is more likely to be a potential risk factor for HPV–cervical cancer progression. Consequences support further attempts to understand the clinical manifestations of neoplasia related to genital infections and gene mutations.

## Introduction

Cervical malignancies are one of the most common disorders in females worldwide. Approximately 86 percent of these cases occur in developing communities, where careHPV, prophylactic screening and a nationally organized program are not commonly available. Lifestyle, environmental conditions, coinfections, genetic patterns and epigenetic characteristics accompanied with HPV genotypes are major risk factors for genital malignancies and disorders [1–3].

Most sexually active women and men are infected at some points in their lives. Additional microbial pathogens such as sexually transmitted infections (STIs) could increase the risk of cervical neoplasia induction [1]. Some single-nucleotide polymorphisms (SNPs) and STI, especially HPV infections, are considered predisposing factors for genital cancerous lesion progression [4, 5].

Methylenetetrahydrofolate reductase, folate-metabolizing enzyme, is a critical component in complex process of folate cycle. SNPs in the MTHFR gene are arguably associated with thrombotic events and may increase or decrease the activity of cancer suppressor genes. Homozygosity for the substitution (C → T) at nucleotide 677 and for the substitution (A → C) at nucleotide 1298 in the MTHFR gene is suggested to be associated with an increased risk of cervical cancer [6–10].

Few studies are found on the association between polymorphisms and STIs related to cervical cancer. The need for identifying genetic susceptibility factors for CC related to HPV and STI is a matter of debate [11]. Therefore, the present study was designed to evaluate the C677T and A1298C mutations of MTHFR gene in cervical intraepithelial neoplasia (CIN) related to HPVs subjects and most common sexually transmitted pathogens such as *Chlamydia trachomatis*, HSV-2 and *Mycoplasma genitalium* in Iranian women suffering from STI coinfections.

## Materials and Methods

### Patients’ Specimens

In this case–control study, 148 women who were referred to Moheb Yas Hospital and other private laboratories in Tehran, Iran with or without genital infections and cervical intraepithelial neoplasia were enrolled in the survey. Forty-seven age- and sex-matched healthy controls were consecutively registered for this study from April 2012 to October 2015. Informed consent was obtained from subjects prior to their registration. Cervical scrapping and genital lesions with known cervical intraepithelial neoplasia (all the diagnoses were confirmed by histopathological examinations), HPV-positive subjects and non-cancerous/non-HPV women as healthy controls were 50, 98 and 47, respectively. These liquid-based cytology samples (LBCs) were transported to a molecular biology laboratory where they were stored in proper condition until genomic analysis.

### DNA Extraction

Extraction of infectious agents and genomic DNAs from LBCs and lesions was performed using a high pure PCR template preparation kit (Roche©, Germany), following the kit protocol. Extracted DNAs were kept at − 20 °C until experiment.

### PCR–RFLP Analysis of MTHFR

Briefly, a 25-μl reaction mixture containing 2x Master Mix PCR (Amplicon^®^, Denmark), 10 pmol/μl of forward and reverse specific primers and 10 μl of the extracted DNA as a template in separate reactions was used for evaluation of MTHFR1298 and 677 mutations. The reactions were amplified in a thermal cycler (PeQlab^®^, UK) by using the following conditions: primary denaturation of 94 °C for 5 min, 32 cycles with 94 °C for 1 min, 63 °C for 1 min and 72 °C for 1 min, plus an additional extension at 72 °C for 10 min. In RFLP analysis, 15 μl of 1298 A > C PCR products were digested with 20 μl of restriction enzyme reaction mixture containing 16 μl of sterile distilled water, 3 of μl blue buffer and 1 μl of MboII (5 U/μL) enzyme (Thermo Scientific^®^). In addition, 10 μl of 677 C > T PCR products were digested with 20 μl of restriction enzyme mixture containing 16 μl of sterile distilled water, 3 μl of Tango buffer and 1 μl of HinfI enzyme (10 U/μL) (Thermo Scientific^®^). The PCR–RFLP products were separated by electrophoresis on a 3% agarose gel, stained by SYBR Safe, and were visualized under ultraviolet light along with a molecular weight marker (50 bp). In a MTHFR 1298, PCR–RFLP was expected to produce 145 bp as undigested, 77, 37 and 29 bp as wild type, 108, 77, 37 and 29 bp as heterozygous and 108 bp as homozygous. In addition, in a MTHFR 677 PCR–RFLP was expected to produce 200 bp as undigested, 200 bp as wild type, 200 and 175 bp as heterozygous and 175 bp as homozygous [12, 13].

### Sexually Transmitted Pathogens Detection

HPV genotyping was performed by a reverse dot blot hybridization diagnostic kit using INNO-LiPA^®^ HPV Genotyping Extra I (Innogenetics ©)/INNO-LiPA^®^ HPV Genotyping Extra II (Fujirebio ©), according to the previously described method. HSV-2, *M. genitalium* and *C. trachomatis* as STIs infections were diagnosed by multiplex quantitative TaqMan real-time PCR which was optimized in our previous study [14, 15].

### Statistical Analysis

The SPSS statistics 23.0 was applied for data analysis. The crude and adjusted odds ratio (OR) and 95% confidence intervals (CI) were calculated with binary logistic regression. In addition, Pearson’s Chi-square test was used for comparing relationship between MTHFR 1298 A > C and 677 C > T genotypes and pathological staging of cervical cancer. *P* value of < 0.05 was considered as significant. Allele frequencies of 1298 and 677 mutations were calculated using Hardy–Weinberg equilibrium. The χ^2^ test was employed to study the deviation from Hardy–Weinberg equilibrium between the observed and expected genotype frequencies in controls.

## Results

The mean age of 148 women with HPV infection and cervical cancer was 37.84 ± 10.55 years, and it was 36.62 ± 9.63 years for 47 healthy controls (non-cancerous/non-HPV) who were recruited in the current case–control study.

**Fig. 1:**
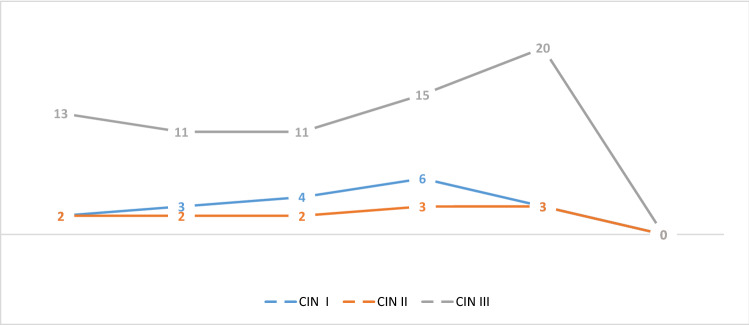
Frequency of MTHFR 1298 and MTHFR 677 genotypes in cervical intraepithelial neoplasia (CIN) subjects

We performed a detailed HPV genotyping and pathogen detection of STIs such as *C. trachomatis*, HSV2 and *M. genitalium* in our previous studies [13, 14]. The details of data are not shown. Subjects infected with high-risk HPV genotypes were at higher risk of cervical cancer (*P* ≤ 0.05). The distribution for the MTHFR A1298C and C677T genotypes in different subjects is shown in Table 1 and Fig. 1. The frequencies of the MTHFR C677T CC, CT and TT genotypes were 52.3, 43.6 and 4.1% among control patients and 48, 52 and 0% among cervical intraepithelial neoplasia cases, respectively. The frequencies of the MTHFR A1298C AA, AC and CC genotypes were 38.9, 41 and 20% among population study and 34, 32 and 34% among cervical intraepithelial neoplasia group, respectively. No significant evidence was found for STI pathogens and these polymorphisms (*P* value ≥ 0.05). The details of MTHFR genotypes prevalence in women with STI pathogens are shown in Table 2. The MTHFR A1298C and C677T genotype frequencies of HPV non-cancerous/non-HPV group were in Hardy–Weinberg equilibrium. [Chi-square was 0.129, 0.21 and 1.43 (*P* value ≥ 0.05).] Comparison of the MTHFR 1298 and 677 polymorphisms between each of the cervical intraepithelial neoplasia subjects, HPV-infected cases and non-cancer/non-HPVs control indicated a significant difference in some genotypes. There was a significant association between the MTHFR 1298 CC polymorphisms (adjusted OR = 3.5, 95% CI = 1.13–10.82, *P* ≤ 0.05) in women with cervical intraepithelial neoplasia and non-cancerous/non-HPV control group.

**Table 1:** The distribution of MTHFR A1298C and C677T genotypes in study population

Clinical subjects		MTHFR 1298 A > C			MTHFR 677 C > T		
		Wild type	Heterozygous	Homozygous	Wild type	Heterozygous	Homozygous
Women without cervical intraepithelial neoplasia (145 cases)	HPV positive (98 cases)	38 (38.7%)	44 (44%)	16 (16.3%)	51 (52.02%)	41 (41.83%)	6 (6.12%)
	Non-HPV/non-cancerous (47 cases)	21 (44.7%)	20 (42.55%)	6 (12.75%)	27 (57.4%)	18 (38.3%)	2 (4.3%)
Women with cervical intraepithelial neoplasia (50 cases)	CIN I = 9 cases, CIN II = 6 cases, CIN III = 35 cases	17 (34%)	16 (32%)	17 (34%)	24 (48%)	26 (52%)	0%
Total		76 (38.9%)	80 (41%)	39 (20%)	102 (52.3%)	85 (43.6%)	8 (4.1%)

**Table 2:** The prevalence of STI pathogens in subjects with MTHFR mutations

Population study		Women with cervical intraepithelial neoplasia (50 cases)			Women without cervical intraepithelial neoplasia (145 cases)						Total	Test of independence
Patients categories		CIN I = 9 cases; CIN II = 6; CIN III = 35			HPV positive (98 cases)			Non-HPV/non-cancerous (47 cases)				
STIs pathogens		*C. trachomatis*	*M. genitalium*	HSV_2_	*C. trachomatis*	*M. genitalium*	HSV_2_	*C. trachomatis*	*M. genitalium*	HSV_2_		
STIs prevalence		7 Positive*	Undetected	Undetected	7	2	1	10	1	0	28	
MTHFR 1298 A > C	Wild type	4^*1^	0	0	3	0	0	6	1	0	14	*χ*^2^ = 0.542 (Chi-square and Fisher’s exact test) (*P* > 0.05)^†^
	Heterozygous	1^*2^	0	0	3	2	0	2	0	0	8	
	Homozygous	2^*3^	0	0	1	0	1	2	0	0	6	
MTHFR 677 C > T	Wild type	2^*4^	0	0	2	2	1	10	0	0	27	*χ*^2^ = 0.481 (Chi-square and Fisher’s exact test) (*P* > 0.05)^†^
	Heterozygous	5^*5^	0	0	5	0	0	0	1	0	11	
	Homozygous	0	0	0	0	0	0	0	0	0	0	

* C. trachomatis-positive cases include CIN I = 1, CIN II = 1 and CIN III = 5

*1CIN I = 1, CIN III = 3

*2CIN III = 1

*3CIN II = 1, CIN III = 1

*4CIN II = 1, CIN III = 1

*5CIN I = 1, CIN III = 4

†There was no significant difference

There was no significant difference between MTHFR 677 genotypes and population study. The heterogeneity, odds ratios and Hardy–Weinberg results are shown in Table 3.

**Table 3:** Heterogeneity, odds ratios and Hardy–Weinberg results in the association between MTHFR polymorphisms and cervical intraepithelial neoplasia

Genotype	Cervical intraepithelial neoplasia *n *= 50	HPV-positive patients *n *= 98	Non-HPV/non-cancerous *n *= 47	OR	95% CI	*P* value	HWE _(control)_	
							*χ*^2^	*P* value
*MTHFR 1298 A *> *C*								
AA	17 (34%)	38 (38.7%)	21 (44.7%)	1.00	Reference		0.129	0.71
AC	16 (32%)	44 (44%)	20 (42.55%)	0.99	0.39–2.47	0.99		
CC	17 (34%)	16 (16.3%)	6 (12.75%)	3.5	1.13–10.82	0.03^†^		
*MTHFR 677 C *> *T*								
CC	24 (48%)	51 (52.02%)	27 (57.4%)	1.00	Reference		0.21	0.64
CT	26 (52%)	41 (41.83%)	18 (38.3%)	1.62	0.72–3.67	0.24		
TT	0	6 (6.12%)	2 (4.3%)	0.00	0.00	0.99		

^†^*P* value ≤ 0.05 (significant)

## Discussion

The association between the MTHFR 1298 A > C and 677 C > T polymorphisms and susceptibility to cervical malignancies and genital disorders has been investigated in the case–control study in Iranian women. We found that HPV 6 (LR), HPV 16 (HR) and *C. trachomatis* were the most common infections among the cervical intraepithelial neoplasia patients and healthy controls. However, HPV 6 is the most common genital infection that might not be an etiological factor for cervical cancer, but is a major cause of genital warts. It seems no previous study has reported the MTHFR A1298C and MTHFR C677T mutations in cervical cancer and STIs pathogens in Iranian women and west Asian regions. Extensive evidence propose that methylenetetrahydrofolate reductase (MTHFR) includes MTHFR A1298C and MTHFR C677T genotypes which may be implicated as potential risk factors in developing cervical cancer. It would appear that gene polymorphisms are implicated in cancer progression and they can be used for screening early stage of malignancies [16–19]. As a result, some genital infections such as high-risk HPVs and STIs are the common causative pathogens of cervical cancer and genital disorders. In addition, multiple co-pathogens appear more likely to be linked and potential risk factors in development of cervical abnormalities, especially in patients with genital infections [14, 20–24].

The function of SNPs in the pathogenesis of cervical cancer coexisting with HPV genotypes and other microorganisms such as *C. trachomatis*, *M. genitalium* and HSV2 in genital disorders has not been extensively studied in developing countries. Multiple sexually transmitted infections are significant causes of infertility, PID, ectopic pregnancy, congenital infections and cancer. Morbidity, mortality and long-term complications of these infections are increasing public health concerns worldwide [1, 14, 17, 18, 25–30]. Several studies have reported that the frequency of SNPs is different in communities and races; for instance, MTHFR 677 TT, CC and CT genotype frequencies in the following populations are as follows: East Asians, India, Mexico, Poland and Netherlands [29–33]. In our study, CC = 27, CT = 11 and TT = 0 were calculated for MTHFR C677T, which are nearly similar to this study such as East Asians, Germany, Poland and India [33], and some of the published articles are not similar to this result which are obtained from the USA, China and Denmark [16, 30–32].

The MTHFR A1298C allele frequency was also appraised in Korea, China and India [33]. In the present study, AA = 14, AC = 8 and CC = 6 were distinguished for the MTHFR A1298C polymorphism, which is similar to the results from India, Korea and China [33] and is in accordance with a Romanian research [10].

The allele’s frequencies can have significant relationship with cervical cancer and genital disorders such as Iran [34], China [16], India and Korea [33]. In some cases, results demonstrated an insignificant correlation between the SNPs and women malignancies. The relevance between MTHFR A1298C and C677T in women suffering from cervical intraepithelial neoplasia and genital infections was significant in MTHFR 1298 CC subjects. The high-risk and low-risk HPV genotypes together with C. tarchomatis, HSV2 and *M. genitalium* were prevalent in cervical intraepithelial neoplasia patients and healthy women (non-cancerous/non-HPV).

## Conclusion

This can be the first study to investigate the association between MTHFR 1298 A > C and 677 C > T in Iranian women suffering from genital disorders. We concluded that some of SNPs were associated with cervical intraepithelial neoplasia related to HPVs and other STI pathogens. We suggest that studies with larger sample size, different *ethnic groups* and use of approved and modern technologies such as whole-genome sequencing are needed to confirm the association between mutations and STIs. Finding a specific molecular biomarker can be helpful in detecting early stage of genital cancers along with diagnosis of sexually transmitted infections.
